# Blame it on the Drug: A Rare Case of Recurrent Doxycycline-Induced Pancreatitis

**DOI:** 10.7759/cureus.29171

**Published:** 2022-09-14

**Authors:** Raghav Bassi, Pranav Prakash, Eason Balakrishnan, George Cockey

**Affiliations:** 1 Internal Medicine, University of Central Florida College of Medicine, Graduate Medical Education/North Florida Regional Medical Center, Gainesville, USA

**Keywords:** antibiotics, acute pancreatitis, clinical case report, drug induced pancreatitis, recurrent acute pancreatitis, oral doxycycline

## Abstract

Doxycycline is a broad-spectrum bacteriostatic antibiotic that belongs to the tetracycline class. It is a relatively safe medication with reported side effects being gastrointestinal symptoms, bone and teeth discoloration, photosensitivity, and renal toxicity. Acute pancreatitis (AP) is an uncommon adverse effect with only a few reported cases in the literature. Despite tetracyclines being labeled as a probable causative agent of drug-induced pancreatitis (DIP), doxycycline has been rarely implicated. Herein we present the case of a 65-year-old patient who developed recurrent doxycycline-induced pancreatitis after she was inadvertently started on the medication for community-acquired pneumonia. The most common causes of pancreatitis were ruled out during her hospital admission and she was subsequently diagnosed with DIP. She was successfully treated with the cessation of the offending agent and with supportive therapy. It is critical that clinicians are aware of the possible association between doxycycline and pancreatitis to further aid in the prompt diagnosis and treatment of this condition.

## Introduction

Acute pancreatitis (AP) is a severe disease affecting approximately 45 per 100,000 people annually [1]. Gallstones and alcohol are the two most common causes comprising 70%-80% of all cases [2]. Other less common etiologies include autoimmune disease, hyperlipidemia, hypercalcemia, post endoscopic retrograde cholangiopancreatography (ERCP), infections, trauma, inflammatory bowel disease, ischemia, and neoplasms [1-2]. An extremely rare etiology of pancreatitis is drug-induced pancreatitis (DIP) with an estimated incidence rate of only 0.1%-2% [1-2]. The World Health Organization has reported 525 drugs linked to AP with a direct causality in 31 drugs. Despite tetracyclines being labeled as a probable causative agent of DIP, doxycycline has been rarely implicated [1]. Doxycycline is a commonly prescribed medication for a wide array of bacterial infections including soft tissue infections, respiratory infections, sexually transmitted infections, and Lyme disease [2]. AP is an uncommon adverse effect with only a few cases reported throughout the literature. Given how commonly doxycycline is prescribed clinically, it is essential for clinicians to be aware of serious adverse effects associated with doxycycline use including DIP. We hope to further build upon existing knowledge and strengthen the association between doxycycline use and pancreatitis by presenting a rare case report of a patient who developed recurrent pancreatitis after taking doxycycline. 

## Case presentation

A 65-year-old woman with a past medical history of DIP, rheumatoid arthritis (RA), and community-acquired pneumonia (CAP) presented to the emergency department with a chief complaint of progressive nausea, fatigue, and epigastric pain radiating to her back for the past two days. She rated the pain as 8/10 in intensity, worse with eating, and occurring persistently throughout the day with no alleviating factors. The patient denied any recent changes except for the fact that she was started on doxycycline three days ago for the treatment of her CAP. The patient also denied any history of smoking, alcohol, insect bites or stings, trauma, or recent endoscopic procedures. 

**Figure 1:**
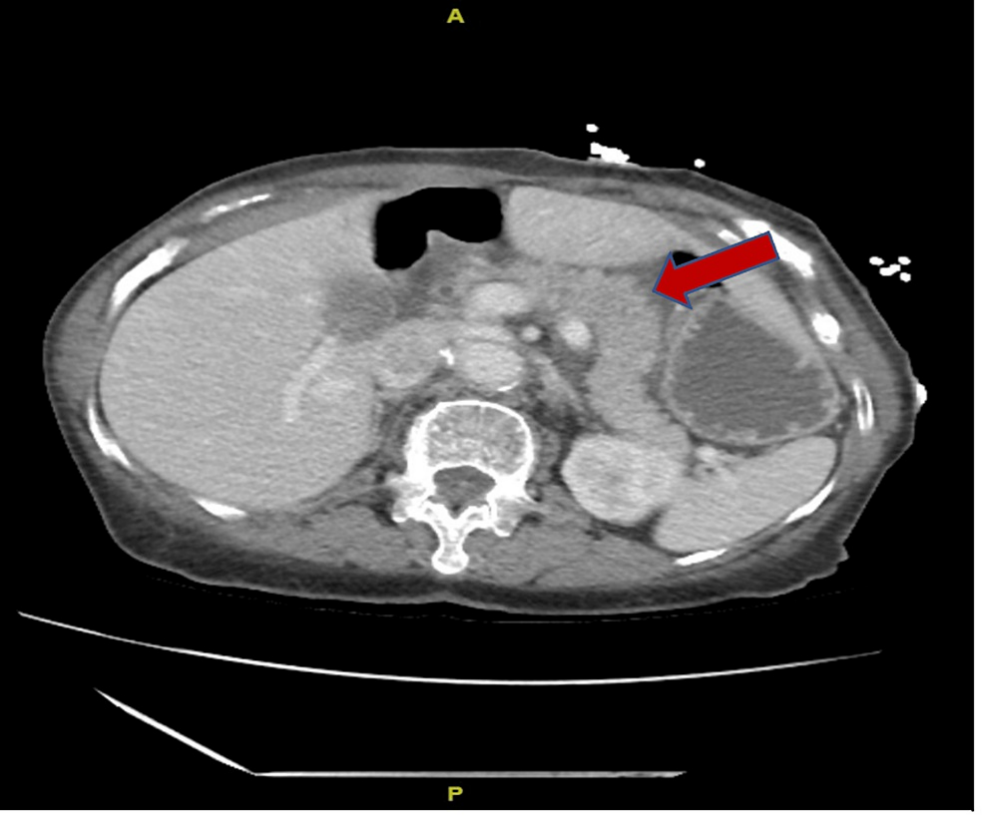
CT of the abdomen and pelvis without contrast revealing evidence of an edematous pancreas with peripancreatic fluid and fat stranding.

**Figure 2:**
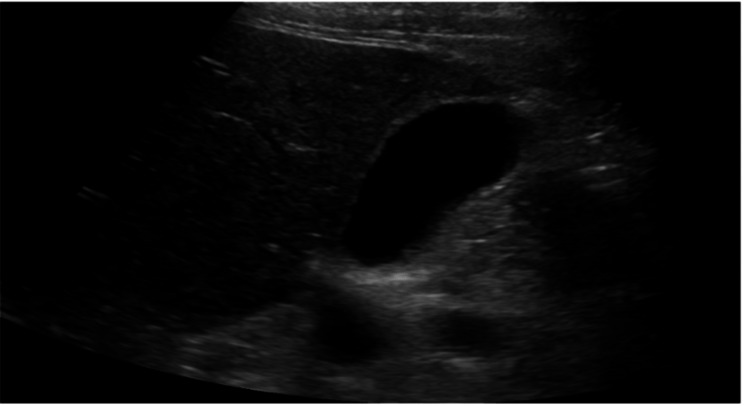
Right upper quadrant ultrasound revealing an unremarkable appearing gallbladder with no evidence of gallstones or wall thickening.

On arrival, she was febrile to 100.4°F and tachycardic. A physical exam was significant for epigastric tenderness with no evidence of abdominal guarding or rigidity. Initial labs were notable for a leukocytosis of 33.9 x 103 µL and lipase of 3431 IU/L with unremarkable serum calcium levels, lactate, liver enzymes, and lipid panel as summarized in Table 1. CT of the abdomen and pelvis revealed peripancreatic fluid and fat stranding consistent with AP (Figure 1). A right upper quadrant ultrasound was then done and it did not show any gallstones or biliary distention (Figure 2). A lipid panel was also ordered to investigate the etiology of her pancreatitis, however, it came back within normal limits. Given her history of RA, an IgG4 level was then ordered to rule out underlying type 1 autoimmune pancreatitis, which was also unremarkable. Blood cultures were also drawn during admission given her fever and leukocytosis; however, there was no microbial growth reported. She was then started on aggressive IV hydration, antiemetics, and pain control. After ruling out the most common etiologies of pancreatitis, doxycycline-induced pancreatitis was diagnosed and doxycycline was stopped. Her lipase improved to 573 IU/L on day 2, and 530 IU/L on day 3 as seen in Table 1. She reported symptomatic relief with the discontinuation of the drug and with supportive care. On further questioning, she reported that her last episode of pancreatitis also occurred in the setting of doxycycline use for the treatment of cellulitis a couple of years ago. She refrained from using doxycycline since that episode, however, she was inadvertently prescribed it and ended up with recurrent symptoms. The patient was then discharged home in improving and stable condition and was instructed to avoid doxycycline use in the future. 

**Table 1:** Pertinent serological labs and tests throughout the hospital stay. BUN, blood urea nitrogen; AST, aspartate aminotransferase; ALT, alanine transaminase; GFR, glomerular filtration rate; MCV, mean corpuscular volume

Labs	Day 1	Day 2	Day 3	Reference range
White cell count (10^3 ^µL)	33.9	23.9	10.3	4.0-10.5
Hemoglobin (g/dL)	12.2	13.3	11.3	13.7-17.5
Hematocrit (%)	40.9	37.4	40.2	40.1-51
Platelets (10^3 ^µL)	254	199	306	150-400
MCV (fL)	98.1	97.4	95	79.0-92.2
Sodium (mmol/L)	140	138	138	136-145
Potassium (mmol/L)	3.7	3.5	4.4	3.5-5.1
Chloride (mmol/L)	109	110	105	98-107
Carbon dioxide (meq/L)	27	23	27	21-32
Glucose (mg/dL)	74	68	124	74-106
BUN (mg/dL)	15	9	18	7-18
Creatinine (mg/dL)	0.64	0.43	0.58	0.6-1.30
GFR (mL/min)	112	178	121	0-120
Calcium (mg/dL)	8.7	8.2	8.5	8.5-10.1
Lipase (units/L)	3283	573	530	73-393
Total bilirubin (mg/dL)	0.4	0.2	0.3	0.2-1.0
AST (units/L)	15	12	7	15-37
ALT (units/L)	12	19	15	13-56
Alkaline phosphatase (units/L)	63	73	57	46-116
Magnesium (mg/dL)	2.1	2.4	2.1	1.8-2.4
Lactic acid (mmol/L)	0.7			0.4-2.0
Triglycerides (mg/dL)	69			0-149
Total cholesterol (mg/dL)	138			<200

## Discussion

Drug-induced pancreatitis is a rare phenomenon with an estimated overall incidence rate of less than 2% of all AP cases [1]. To date, 31 different medications have been identified as definite causes of DIP with the most common being: mesalazine, azathioprine, and simvastatin [2]. Most of our understanding of this disease process is based on case reports identifying a causal relationship between pancreatitis and a potential offending agent in the absence of other common etiologies. The lack of clinical features specific to DIP makes it challenging to diagnose and further adds to its complexity. Tetracyclines have been implicated as a definite cause [3] of pancreatitis and are classified under class 1b of the Badalov classification of DIP [4]. Doxycycline, however, remains an uncommon cause of DIP. To our knowledge, there have only been six reported cases of doxycycline-induced pancreatitis in the literature with this being one of the first reported cases of recurrent pancreatitis [5]. 

The most common causes of AP are alcohol use and gallstones comprising 70%-80% of all cases followed by hypertriglyceridemia and procedures such as endoscopic retrograde cholangiopancreatography (ERCP) [6]. Other less common etiologies are usually patient-specific and include autoimmune causes, infections, and trauma. As seen in the case presentation above, the most common etiologies of pancreatitis were initially ruled out as she had no history of significant alcohol use or recent endoscopic procedures such as ERCP. A right upper quadrant ultrasound was negative for gallstones and serological testing demonstrated normal lipid levels. Furthermore, she denied any recent tick or insect bites, travel, or trauma. Autoimmune pancreatitis was also considered as a possible differential given her history of RA, however, she tested negative for IgG4 antibody. Thus, we were able to eliminate the above as causes of her AP. However, her recent use of doxycycline for the treatment of her CAP just three days prior to the onset of her symptoms raised suspicion for DIP. 

 Our patient developed pancreatitis within three days of exposure to doxycycline and her symptoms resolved promptly on discontinuation of the medication. DIP is diagnosed after excluding common etiologies of pancreatitis, followed by a clinical improvement after cessation of the offending agent as seen in the case above. However, in addition to doxycycline discontinuation, she was also concurrently treated supportively for her AP with bowel rest, IV fluids, antiemetics, and pain control raising the question of whether the etiology of her pancreatitis was indeed drug-induced or rather idiopathic. 

Our patient’s history of a similar episode of AP two years ago in the setting of doxycycline use further favors the diagnosis of DIP as the most likely etiology. She also scored eight on the Naranjo Adverse Drug Reaction scale revealing that there was a probable adverse reaction to doxycycline furthering our suspicion of DIP. This case is particularly unique given that there have been no documented cases of recurrent pancreatitis due to doxycycline use to our knowledge. 

Various theories have been proposed to explain DIP since it is believed that each class of medication has a unique mechanism [4]. It is postulated that tetracyclines can cause DIP either through a toxin-mediated effect of an unknown metabolite or a direct toxic effect on the pancreas due to the supratherapeutic biliary concentration of tetracyclines [7]. Pharmacological trials with tetracyclines have shown the biliary concentration of minocycline to be 10 times higher than the serum concentration and similar results were observed with tigecycline [7]. These findings can help explain doxycycline-mediated pancreatitis given that minocycline and doxycycline are both group 2 tetracyclines and have similar pharmacokinetic profiles [8]. However, more research is needed to explain the exact pathophysiology of doxycycline-induced pancreatitis.

## Conclusions

Doxycycline is a commonly prescribed antibiotic and in rare instances, it can cause DIP as seen in the case above. Clinicians should be aware of this potential adverse effect and should maintain a high degree of suspicion in patients who recently received doxycycline therapy and are presenting with features suggestive of pancreatitis. DIP is a diagnosis of exclusion with alcohol, gallstones, hypertriglyceridemia, and underlying electrolyte abnormalities being ruled out first as possible etiologies of the patient's pancreatitis. Management constitutes early identification and cessation of the offending agent resulting in the resolution of symptoms.
